# Semantic discrimination impacts tDCS modulation of verb processing

**DOI:** 10.1038/s41598-017-17326-w

**Published:** 2017-12-07

**Authors:** Valentina Niccolai, Anne Klepp, Peter Indefrey, Alfons Schnitzler, Katja Biermann-Ruben

**Affiliations:** 1 Institute of Clinical Neuroscience and Medical Psychology, Medical Faculty, Heinrich-Heine-University, Duesseldorf, Germany; 2 Institute for Linguistics and Information Science, Heinrich-Heine-University, Duesseldorf, Germany; 3 Donders Institute for Brain, Cognition and Behaviour, Radboud University, Nijmegen, Netherlands

## Abstract

Motor cortex activation observed during body-related verb processing hints at simulation accompanying linguistic understanding. By exploiting the up- and down-regulation that anodal and cathodal transcranial direct current stimulation (tDCS) exert on motor cortical excitability, we aimed at further characterizing the functional contribution of the motor system to linguistic processing. In a double-blind sham-controlled within-subjects design, online stimulation was applied to the left hemispheric hand-related motor cortex of 20 healthy subjects. A dual, double-dissociation task required participants to semantically discriminate concrete (hand/foot) from abstract verb primes as well as to respond with the hand or with the foot to verb-unrelated geometric targets. Analyses were conducted with linear mixed models. Semantic priming was confirmed by faster and more accurate reactions when the response effector was congruent with the verb’s body part. Cathodal stimulation induced faster responses for hand verb primes thus indicating a somatotopical distribution of cortical activation as induced by body-related verbs. Importantly, this effect depended on performance in semantic discrimination. The current results point to verb processing being selectively modifiable by neuromodulation and at the same time to a dependence of tDCS effects on enhanced simulation. We discuss putative mechanisms operating in this reciprocal dependence of neuromodulation and motor resonance.

## Introduction

The assumption that cognition is grounded in simulation processes^1,2^ implies a cross-talk between action-related language and neurophysiological motor mechanisms. Cortical motor engagement accompanying the processing of verbs and action-related sentences has indeed been detected by means of blood-oxygen-level dependent (BOLD) signal^3–5^, event related potentials/fields (ERPs)^6–8^, and neural oscillatory activity^9,10^. Also, behavioural measures of verbal-motor interaction such as priming and interference effects consistently hint at shared brain resources between linguistic and motor processes (for a review see^11^). Complementary neurophysiological investigations confirm that priming and interference exert a modulatory effect on cortical motor activation^12–14^. Yet, some studies on cortical motor lesions point to a lack of impairment in action word processing and to a possible involvement of other brain areas^15,16^ thus raising questions on the causal relevance of cortical motor activation for action-related linguistic understanding.

To tackle this issue, a number of studies have applied transcranial magnetic stimulation (TMS) of the motor cortex while participants processed action verbs and sentences. Overall, results point to a word-dependent modulation of cortical motor excitability as measured with motor evoked potentials (MEPs) and reaction times. Interestingly, abstract action knowledge as elicited by motor-related pictures^17^ and athletes’ surnames^18^ can also affect response latency and cortical MEPs in the primary motor cortex. While there may be a dissociation between cortical motor excitability elicited by abstract action knowledge and observation of real actions^18^, results from TMS studies indicate an instrumental role of the motor cortex in language understanding. However, an inconsistent scenario concerning the direction of this modulation emerges. Some investigations showed decreased MEPs and/or longer reaction times suggesting inhibited cortical motor activation^19–22^, while others showed increased MEPs and/or shorter reaction times^23–25^ indicating cortical motor facilitation. As for inconsistencies among single pulse TMS studies, these may depend on whether MEP recording accompanied word onset^19,24,26^ or took place at subsequent time-points (i.e., between 170 and 500 ms after word onset^22,25^. Although other methodological differences such as types of protocols (e.g., repetitive versus single pulse TMS) and stimuli may also to some extent explain inconsistencies, opposite TMS effects have been observed despite applying the same paradigm and stimuli^19,26^. To clarify the role of increased versus decreased cortical excitability in verbal processing an alternative approach can be applied that bi-directionally manipulates cortical motor excitability.

Transcranial direct current stimulation (tDCS) induces changes in the membrane permeability and accomplishes a moderate shift of cortical excitability^27^. This technique offers some advantages over TMS. First, it allows the investigation of bi-directional effects of stimulation by depolarising and hyperpolarising the cellular membrane. Anodal and cathodal stimulation of the hand-related motor area led to motor cortical excitation and inhibition respectively^27,28^. Significant reduction in gamma-aminobutyric acid (GABA) concentration corroborates the excitatory effect of anodal stimulation of the hand-related cortical motor area^29^. In the context of linguistic processing, tDCS has often been applied to frontal, parietal and temporal areas (see^30,31^ for reviews). By contrast, tDCS of the cortical motor area has seen applications in motor observation^32^. As for tDCS application to the motor cortex in linguistic paradigms, only a few studies were conducted^33–35^. The present study aimed at filling this gap by determining the bi-directional modulatory effect of stimulation on the verbal-motor interface in healthy individuals and thus further characterizing the functional contribution of the motor system to linguistic processing.

An important characteristic of tDCS is that, differently from TMS, it modulates spontaneous cortical activation instead of disrupting it^36^. Although it induces cortical noise like TMS, the dependent neural activity is strongly influenced by the state of the system, which is mainly determined by the task^36,37^. Anodal tDCS induces firing of neurons that are near threshold: if neurons are not influenced by the task, they will be far from the threshold and will less likely discharge^37^. This makes tDCS particularly suited to address the role of semantic processing for cortical motor activation. In general, when semantic access is irrelevant for task completion, hand related expressions do not induce significant motor resonance^11^. Conversely, motor activation seems to depend on deeper understanding of action verbs as required by a semantic task^38^. Results from studies on abstract action knowledge based on the relationship between personal names and athletes’ motor skills suggest that the strength of the association between name and motor content needs to rise above some level of consolidation for the process of embodying in the motor system to take place^39^. By varying task requirements, the depth of semantic processing was shown to affect verbal-motor interference^40^ and priming^41^, possibly by modifying the amount of recruitment of the motor system. In the current study we addressed the synergies between tDCS and semantic processing depth, defined as the individual accuracy in a verb categorization task. Both the effect of cortical motor stimulation on verb processing and the role played by semantic processing depth in tDCS-induced modulatory effects on reaction times were examined. A dual, double-dissociation task was applied in which a) a verb had to be semantically categorized as concrete versus abstract and b) in case of a concrete verb, hand/foot responses had to be given to a verb-unrelated prompt (a shape with rounded or pointed corners). This priming paradigm has the advantage of disentangling hand from foot-related verbal and motor contributions: responses from both effectors follow the same stimuli thus allowing the attribution of possible differences to priming. On the other hand, this task does not allow direct measure of response latencies to verb presentation: responses are prompted by the shape and not by the verb thus precluding contrasts in reaction time between different verb categories (e.g., concrete versus abstract verbs, or hand versus foot verbs). Online tDCS of the left hemispheric hand knob was expected to selectively affect processing of hand- but not foot-related verbs. As tDCS does not seem to affect simple motor hand reactions^42,43^, no tDCS effect on hand reaction times was anticipated. Capitalizing on the stronger reliability of the sham stimulation in tDCS compared to TMS paradigms^44–46^, we applied it as a control condition in a double-blind cross-over design. Planned comparisons between verum and sham stimulation were expected to result in opposite effects of anodal and cathodal tDCS on behavioural measures.

## Methods

### Participants

Twenty monolingual German native speakers (10 females, mean age = 23.8 ± 6 SD) took part in the study. The sample size was based on a previous investigation with the same paradigm showing that behavioural effects emerged already with 15 participants^41^. All subjects were right-handed, with an average laterality quotient of 92.3% (SD = 16.2%; Edinburgh Handedness Inventory^47^); 15 subjects were right-footed, 4 showed no clear side preference and 1 was left-footed (Lateral Preference Inventory^48^). The subjects had normal or corrected-to-normal vision and none made use of neuro-modulatory medications. Exclusion criteria were history of serious medical, neurological, or psychiatric illnesses, severe head trauma, personal or family history of epilepsy, metal implant in the head/neck region, pacemaker implantation and pregnancy. Participants provided written informed consent prior to each measurement and received financial compensation for their participation. The study was in accordance with the Declaration of Helsinki and was approved by the local ethics committee of the Medical Faculty at the Heinrich Heine University in Duesseldorf (study number 3400).

### Materials

German disyllabic infinitive verbs describing actions executed with the upper extremities (hand verbs), with the lower extremities (foot verbs), and actions in which no body part was involved (abstract verbs) were visually presented. The three conditions underwent a matching procedure for the parameters word length, frequency, imageability and familiarity to properly select stimuli. Word familiarity and imageability were assessed by means of two rating scales (each n = 30, see^8,9^ for further details) and word frequency was derived by the Leipzig corpora collection^49^. Each condition finally included 48 verbs, which were also used in previous studies^8,9^, repeated twice. Hand and foot verbs did not differ in familiarity, length, imageability and frequency (all p > 0.283).

The prompt stimulus consisted of 12 different star-like shapes, each of which could have rounded or pointed corners (further details in^41^). Presentation software (version 14.9, Neurobehavioral Systems, Albany, California, USA) was used to display the stimuli. The space bar of a standard computer keyboard and a foot-pedal (Foot Switch SW3-M, PCsensor) were used as hand and foot response device, respectively.

### Procedure

**Figure 1:**
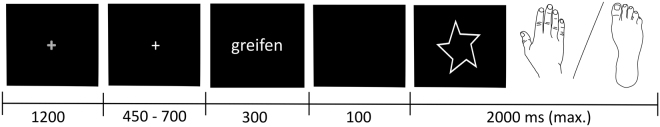
Task design. In the case of a concrete verb (e.g.,“greifen” = “to grab”), participants had to respond to a shape by pressing a hand button or a foot pedal depending on the shape’s corners (here: pointed corners).

The experimental design consisted in a double-dissociation paradigm based on a Go-NoGo task that required semantic processing. For each verb presentation, participants were required to identify whether the verb was concrete or abstract: concrete verbs defined the Go-condition and participants had to respond later on with the right hand or with the right foot depending on the type of corners of a prompt shape (rounded/pointed, Fig. 1) as soon as the prompt occurred. Abstract verbs represented a NoGo cue. Each verb was followed once by each prompt type. The association between the shape’s rounded/pointed corners and the response effector was counterbalanced across subjects and kept stable across three sessions (see below). Each trial began with a central fixation cross displayed on a black background for a jittered interval between 1200 and 1700 ms. Then a word in white font appeared centrally and remained on the screen for 300 ms followed by 100 ms black screen. Afterwards, a shape with either rounded or pointed corners was centrally presented for a maximal time of 2 s. Hand or foot responses ended the shape presentation and initiated the following trial. The experiment was split into two blocks each including 144 trials; a break lasting one minute separated the two blocks. Overall the measurement lasted about 18 minutes and each subject underwent three sessions on three different days; the sessions were one week apart and generally at the same time of the day. A total of 18 stimuli different from those of the main study were used in practice trials preceding the main experiment and each word was randomly followed by both types of shapes. The training lasted about 4 minutes in the first session and 1 minute in the following two sessions. Participants were not informed about the aim of the experiment being related to hand and foot verbs.

### TMS

Transcranial magnetic stimulation was applied to localise the hand-related primary motor cortex of the left hemisphere by determining the individual resting motor threshold. After about 10 minutes of relaxed sitting, participants received single pulse TMS, delivered by a standard eight-shaped coil (MC-B70) connected to a stimulator (Medtronic MagPro, Minneapolis, USA). This was tangentially placed on the scalp of the participant, with the handle pointing backwards and laterally at about 45 degree away from the midline. The target site was marked with a marker pen on the skin of the participant’s head.

### tDCS

Two saline-soaked sponge electrodes were placed on the head of the participants after locally cleaning the skin surface with alcohol and applying scrubbing gel (Abralyt HiCl). One smaller tDCS electrode (3 × 3 cm) was positioned on the left hemispheric hand motor cortex and the other larger electrode (7 × 5 cm) was located on the contralateral supra-orbital region. The use of a smaller electrode was aimed at narrowing the stimulated area around the hot spot^50^. A current intensity of 0.75 mA was applied using a battery-driven stimulator system (NeuroConn, Ilmenau, Germany) and impedance was kept below 11 kOhm. Stimulation parameters were in accordance with current safety guidelines of transcranial brain stimulation^45,51^. Online anodal, cathodal or sham stimulation was applied during the main experiment, starting after the practice trials. Stimulation was pseudorandomly delivered to the participants in a double-blind, cross-over design so that each subject received each type of stimulation once (anodal, cathodal, and sham). To mimic the sensation of verum stimulation in the sham condition the current was ramped up for 10 seconds and then immediately ramped down for 10 seconds. At the end of each session a debriefing questionnaire was administered to assess the participant’s blindness to the type of stimulation. On average each subject correctly guessed only one session out of three, i.e. guessing was at chance level and thus indicated effectiveness of the blinding procedure. 31.5% of the subjects correctly guessed the anodal and the sham condition and 36.8% the cathodal condition. Data from the cathodal condition of one subject and from part of the sham condition of another subject were not available due to technical problems.

### Analysis

Log-transformed reaction times for correct responses and shape-response task accuracy were analysed using linear mixed models^52^. This method is advantageous due to its sensitivity to differences among individual subjects, robustness to unequal sample sizes (unbalanced designs can be analysed without eliminating or replacing data-points), and for reducing variance by accounting for item-related differences in performance^53^, which optimizes generalization over participants and word samples. To capture accuracy related to word processing the discrimination parameter d-prime (d’) was assessed for the single sessions of each participant. This specifically referred to the accuracy of the first (semantic) part of task, where concrete versus abstract verbs had to be identified; the accuracy of responses to shapes was not concerned. d’ was calculated as a difference between the normalised rate of hits to concrete words (i.e., responses from any effector following concrete words) and the normalised rate of false alarms to abstract words (i.e., responses from any effector occurring although they should have been inhibited). In the cases of perfect accuracy, which results in infinite d’ values, the accuracy was estimated as half way between the best value of the corresponding tDCS condition and 100% accuracy to preserve the order of the subject performance ranking. Reaction times exceeding two standard deviations within each session and response effector were eliminated. This was motivated by the effect that learning and responses with different body parts have on reaction times^54^ and accuracy^14^. Data points from one session of one subject were excluded because the related d’ value exceeded two SD across all d’ values.

For reaction time analysis, the following four factors were employed in the linear mixed model using the package lme4^55^ run on R^56^: tDCS condition (anodal, cathodal, and sham), response effector (hand, foot), verb type (hand, foot) and semantic discrimination performance expressed as median split d’ values (high, low). High and low d’ indicated higher and lower performance, respectively. d’ values were calculated per subject and per single session, so each subject could score differently depending on the performance in each of the three sessions. As literature evidence shows inconsistencies in the presumed opposite modulatory effects of anodal and cathodal stimulation (see Discussion section), we opted for the sham condition as a neutral and more adequate control condition. We a priori sum contrasted all fixed effects, thus resulting in the following planned comparisons for the tDCS factor: anodal versus sham and cathodal versus sham stimulation. The main effects and interactions of the four factors were specified in the model as fixed effects. Crossed random effects for participants and items were applied. Random effects for participants included random intercepts and random slopes of the four main effects and their interactions starting with a maximal, design-driven random effects structure^57^. The converging model with the maximal random effects structure included random intercepts for participants and items as well as random slopes of the four main effects for participants. P-values were computed via Wald-statistics approximation. Post-hoc contrasts of verb type by effector interactions were done for each effector separately to exclude an influence of the different latency of hand versus foot responses. The post-hoc tests were performed with lsmeans^58^ adopting the Tukey method for multiple comparisons; the confidence level was set at 0.95.

**Table 1:** Formula and statistical results from the mixed model analysis of reaction times (a-tDCS = anodal vs. sham; c-tDCS = cathodal vs. sham). P-values are computed via Wald-statistics approximation and significant p-values are shown in bold.

Formula: log-rt ~ DCS*verb*effector*d‘ + (1 + verb + tDCS + effector + d‘|subject) + (1|item)			
Fixed parts	*Estimate*	*Std. Error*	*p-value*
(Intercept)	6.521	0.028	**<0.001**
a-tDCS	0.003	0.015	0.828
c-tDCS	0.000	0.017	0.991
verb	−0.001	0.006	0.830
effector	−0.060	0.010	**<0.001**
d’	−0.034	0.017	**0.045**
a-tDCS × verb	0.002	0.003	0.554
c-tDCS × verb	0.002	0.003	0.389
a-tDCS × effector	−0.001	0.003	0.703
c-tDCS × effector	0.001	0.003	0.844
verb × effector	0.009	0.002	**<0.001**
a-tDCS × d’	0.018	0.011	0.079
c-tDCS × d’	−0.013	0.013	0.316
verb × d’	0.001	0.003	0.642
effector × d’	−0.000	0.003	0.951
a-tDCS × verb × effector	0.002	0.003	0.353
c-tDCS × verb × effector	0.001	0.003	0.624
a-tDCS × verb × d’	−0.001	0.003	0.611
c-tDCS × verb × d’	0.008	0.003	**0.006**
a-tDCS × effector × d‘	0.004	0.003	0.198
c-tDCS × effector × d‘	−0.002	0.003	0.512
verb × effector × d’	0.001	0.002	0.777
a-tDCS × verb × effector × d’	−0.002	0.003	0.541
c-tDCS × verb × effector × d’	−0.001	0.003	0.716

For shape-response accuracy analysis, main effects and interactions between tDCS condition, response effector, and verb type were specified as fixed effects in a binomial logit model^59^; random effects for participants included random intercepts. As the logit model predicts the probability of a particular outcome, only correct responses for concrete verbs were included in the analysis.

## Results

Subjects correctly performed the semantic categorization task as indicated by an average accuracy of 91.1% (SD = 9.05) in inhibiting responses after abstract verbs and of 93.7% (SD = 10.49) in responding after concrete verbs.

**Figure 2:**
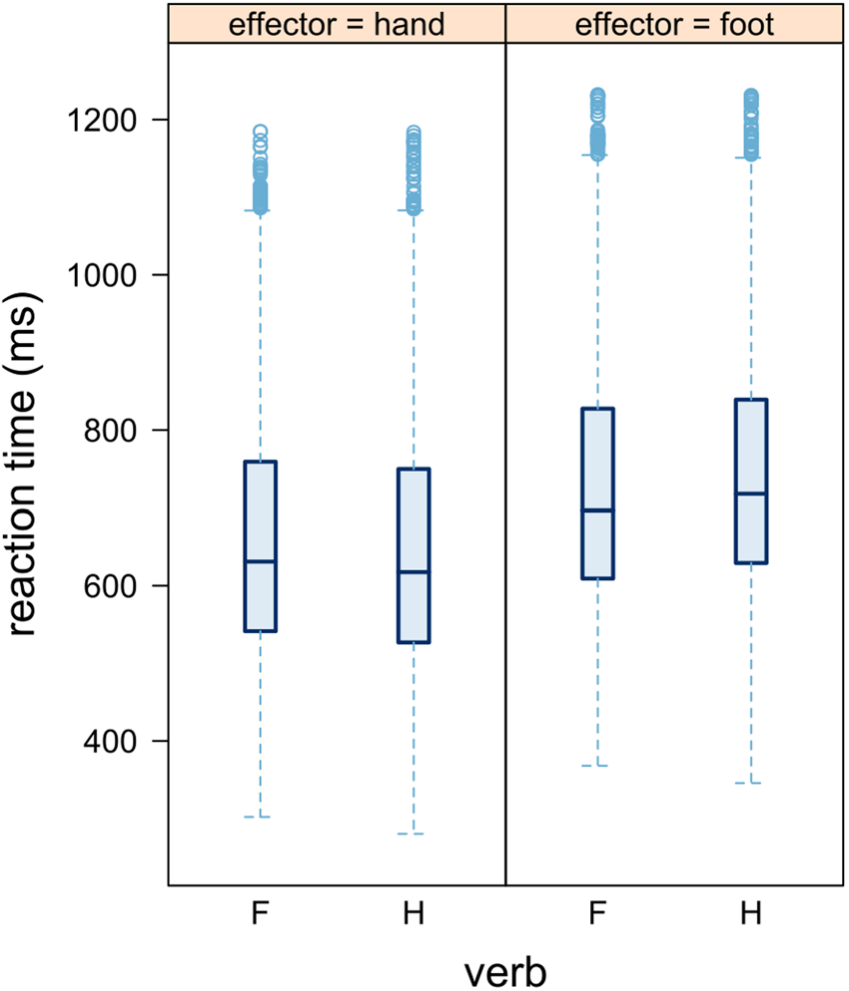
Averaged raw reaction times for each response effector following hand (H) and foot (F) verbs; the horizontal line shows the median, the box indicate the 25^th^ and 75^th^ percentile and whisker limits are at 1.5 interquartile range.

**Figure 3:**
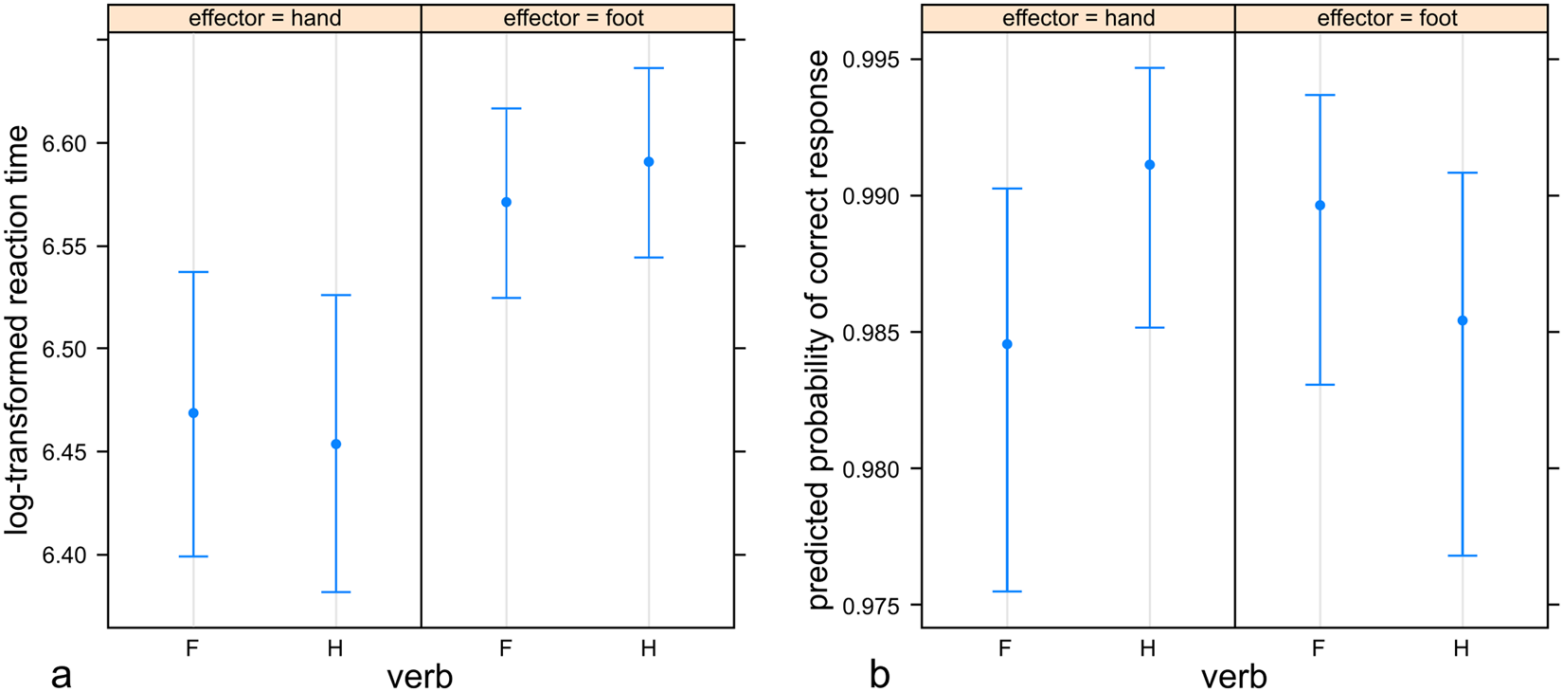
Priming effect across tDCS conditions on reaction times (**a**) and shape-response accuracy measures (**b**). Estimates and confidence intervals for verb type (H = hand, F = foot) and response effector.

**Figure 4:**
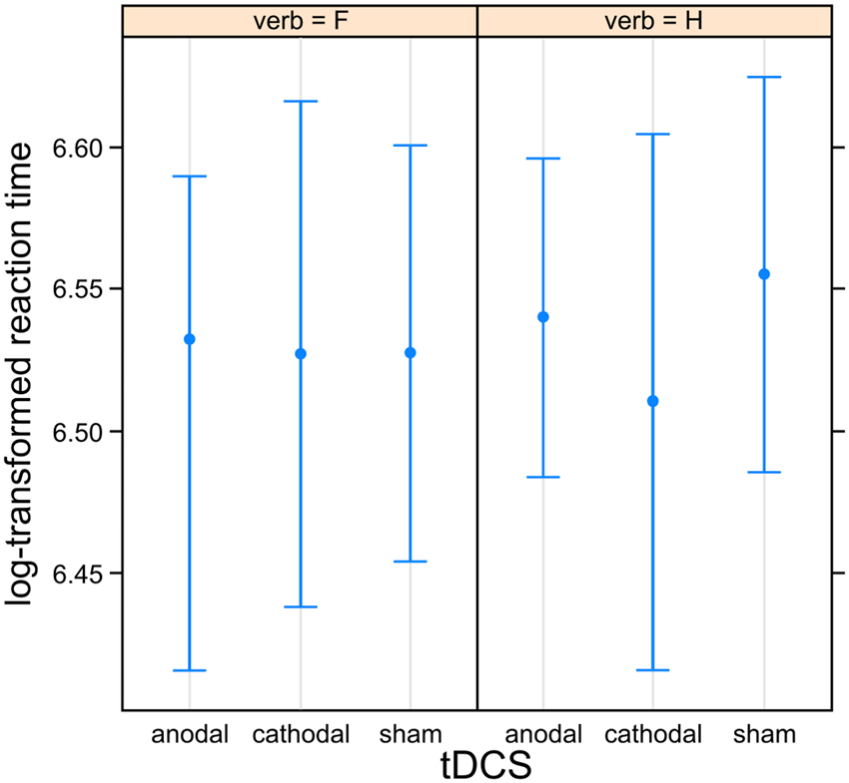
Estimates and confidence intervals for tDCS and verb type (H = hand, F = foot) on logarithmically transformed reaction times for the subgroup with high semantic discrimination.

Response effector showed a significant main effect consisting in faster hand than foot responses (p < 0.001; Table 1). Semantic discrimination as measured for each participant in each session showed a main effect consisting of significantly faster responses for high versus low verb discrimination accuracy (d’; p = 0.045). A significant interaction between verb type and response effector indicated a facilitation effect consisting in shorter reaction times for congruent verb-effector pairs (p < 0.001; Figs 2 and 3a). Yet, post-hoc analysis did not result in significant differences between reaction times for hand versus foot verbs (all p > 0.883). Importantly, there was a significant interaction between cathodal versus sham stimulation (c-tDCS), verb type and semantic discrimination (p = 0.006). To disentangle this 3-way interaction, data subsets for high and low semantic discrimination were created. The main effects and the interactions between tDCS and verb as well as between verb and effector were specified in the model as fixed effects. Random effects for participants included random intercepts and random slopes, starting with a maximal random effects structure. The converging model with the maximal random effects structure included random intercepts for participants and items as well as random slopes of tDCS main effect and of verb-effector interaction for participants (Table 2). A significant main effect of response effector (p < 0.001) and a significant interaction of verb with effector pointing to a priming effect were confirmed in both data subsets (p = 0.023 and p = 0.011; Table 2). Crucially, while the cathodal versus sham planned contrast interacted significantly with verb in the high semantic discrimination subset (p = 0.003), it did not do so in the low semantic discrimination subset (p = 0.190). This significant interaction between cathodal versus sham stimulation and verb type consisted in faster responses to prompts following hand verbs in the cathodal compared to the sham condition (Fig. 4) independently from response effector, while no tDCS effect was observed for foot verbs.

**Table 2:** Formula and statistical results from the mixed model analysis of reaction times in the subsets with high (left) and low (right) semantic discrimination (d′). P-values are computed via Wald-statistics approximation and significant p-values are shown in bold.

Formula: log-rt ~ tDCS*verb + verb*effector + (1 + tDCS + verb*effector|subject) + (1|item)						
**Fixed parts**	high d’			low d’		
	*Estimate*	*Std. Error*	*p-value*	*Estimate*	*Std. Error*	*p-value*
(Intercept)	6.532	0.034	**<0.001**	6.532	0.031	**<0.001**
a-tDCS	0.004	0.012	0.748	−0.031	0.027	0.257
c-tDCS	−0.013	0.020	0.506	0.048	0.034	0.158
verb	−0.003	0.007	0.627	−0.001	0.006	0.911
effector	−0.045	0.010	**<0.001**	−0.070	0.011	**<0.001**
a-tDCS × verb	−0.001	0.004	0.753	0.003	0.004	0.490
c-tDCS × verb	0.012	0.004	**0.003**	−0.005	0.004	0.190
verb × effector	0.009	0.004	**0.023**	0.008	0.003	**0.011**

**Figure 5:**
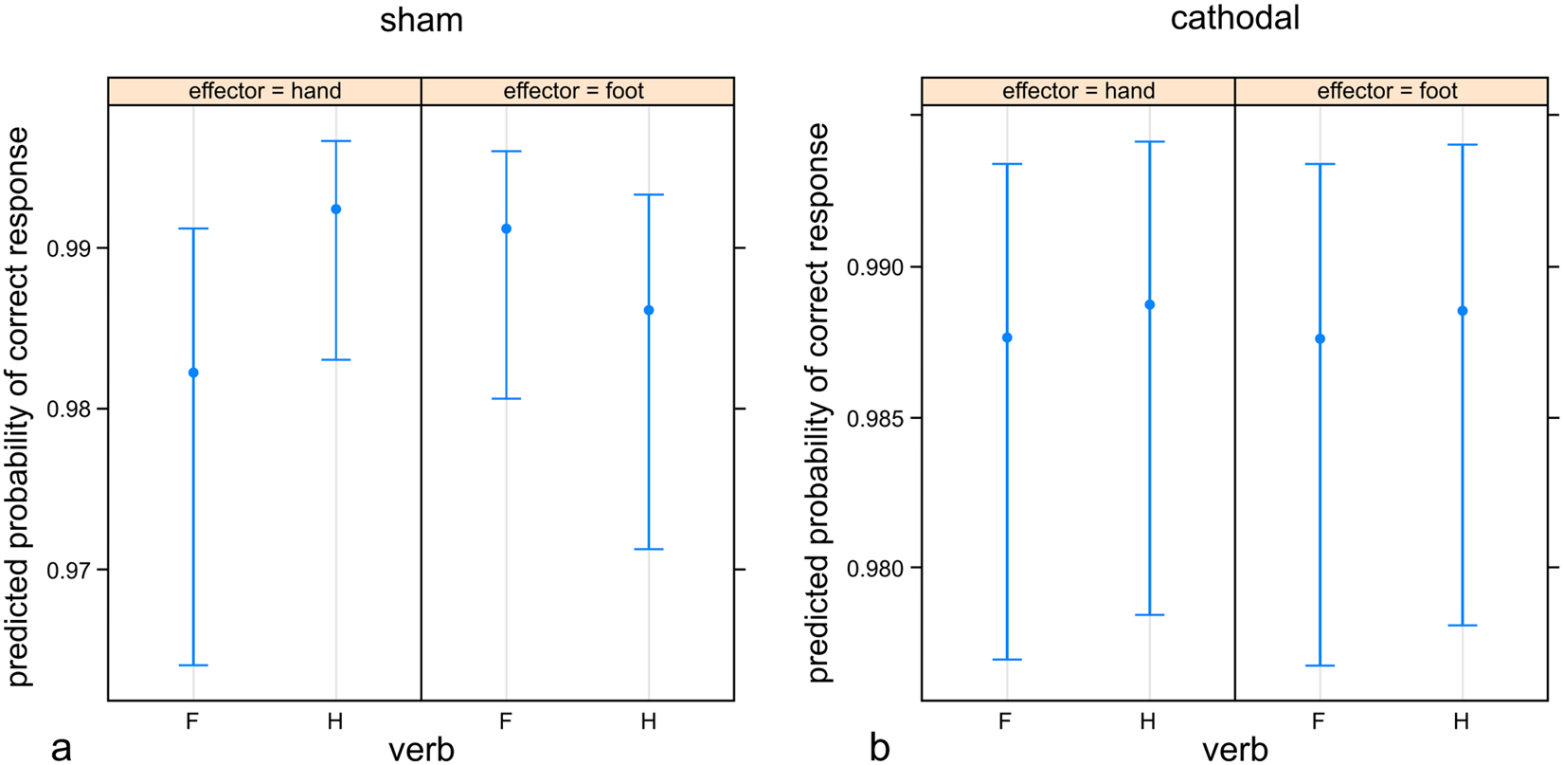
Estimates and confidence intervals for verb type and response effector in the sham (left) and cathodal (right) condition. Note the absence of priming in accuracy measures with cathodal stimulation.

For shape-response task accuracy, a significant interaction of verb with effector (p = 0.004) showing higher accuracy for congruent verb-response pairs pointed to a facilitation effect (Fig. 3b, Table 3). Post-hoc analysis resulted in significantly more accurate hand responses for hand verb primes (p = 0.028) and a trend for significantly more accurate foot responses for foot verb primes (p = 0.059). Also, the interaction between cathodal versus sham tDCS, verb type, and response effector approached significance (p = 0.050). To follow up this interaction, models including the main effects and the interaction of verb with response as fixed effects and random intercepts for participants were separately applied to the cathodal and the sham condition: while the interaction remained significant in the sham condition (p = 0.003), it was not significant in the cathodal condition (p = 0.987) indicating a loss of the priming effect regarding accuracy (Fig. 5). Follow-up analysis showed the presence of a significant priming effect also in the anodal condition (p = 0.013), with p-values surviving post-hoc Bonferroni multiple comparisons correction.

**Table 3:** Formula and statistical results from the mixed model analysis of shape-response accuracy. Significant p-values are shown in bold.

Formula: accuracy ~ tDCS*verb*effector + (1|subject)			
**Fixed parts**	*Odd ratios*	*Std. Error*	*p-value*
(Intercept)	82.080	0.209	**<0.001**
a-tDCS	1.156	0.114	0.204
c-tDCS	1.194	0.116	0.128
verb	1.055	0.078	0.499
effector	1.024	0.078	0.767
a-tDCS × verb	0.967	0.114	0.766
c-tDCS × verb	0.982	0.115	0.876
a-tDCS × effector	1.072	0.114	0.544
c-tDCS × effector	0.981	0.115	0.869
verb × effector	1.254	0.078	**0.004**
a-tDCS × verb × effector	1.145	0.114	0.236
c-tDCS × verb × effector	0.798	0.115	0.050

## Discussion

Beyond a predictable advantage of hand over foot reaction times likely due to nerve conduction speed^54^ and to practice, a priming effect of verb type on response latency and accuracy emerged, whereby the latter was statistically mainly expressed by hand verb primes. Together with previous results showing limb-specific facilitation in response latency with a similar task paradigm^60^, the present findings indicate a somatotopically localised engagement of hand and foot motor cortices in processing body-related verbs as hand/foot-verbs seem to recruit the related hand/foot motor area in a faster and more accurate way. Motor simulation^1,2^ appears a plausible mechanism underlying this facilitation effect. Crucially, in case of high semantic discrimination cathodal stimulation of the hand motor cortex accelerated responses for hand but not for foot verb primes.

Previous studies have shown interference effects to precede facilitation effects by varying the time-lapse between verb presentation and action onset. A systematic review of studies focussing on hand-related expressions showed that single word presentation of hand verbs delays hand responses in early time-windows (up to 400 ms) and subsequently facilitates them between 450 and 750 ms^11^. This agrees with the neurocomputational perspective that the time-window up to half a second after verb onset is accompanied by neuronal decrease of firing rate in the premotor cortex, whereas a facilitation time-window from half a second to about a second is related to more rapid neural reaction^61^. Early interference and subsequent facilitation thus constitute a temporal dynamics with which online stimulation as applied in the present study interacts.

A plausible mechanism underlying faster responses for hand verb primes is that the cortical inhibitory effect of cathodal stimulation (see Introduction) selectively decreased cortical motor activation induced by hand verb processing and by hand response preparation. Although the present task design does not provide direct measures of interference, task timing and reaction time improvement suggest reduced interference between verb processing and motor preparation as triggered by concrete words. Differential engagement of hand and foot motor areas in concrete versus abstract verb processing has already been found around 200 ms after word onset^9^, thus conceivably leading to early response preparation in the present design. From a neurophysiological perspective, concurrent linguistic and motor (preparation) processes appear to compete for common neural resources. This is suggested by reduced cortical excitability^19^ and decreased motor-related activity accompanying the overlap between hand/foot-related word processing and congruent limb action execution/preparation^12–14,62^. Accordingly, a cathodally induced decrease in cortical excitability may have reduced competition by shifting activation dependent on hand verb processing and hand response preparation to a below-threshold state. Reduced interference thus possibly led to an earlier onset of priming and faster hand responses. In line with our findings, the application of theta burst TMS to the left hemispheric hand-related motor cortex, a technique known to reduce cortical excitability^63^, shortened hand reaction times to hand verbs compared to right hemispheric stimulation^64^. Altogether, results suggest that externally-induced decline in cortical excitability may disrupt verbal-motor interference and allow faster cortical recovery processes.

Cathodal stimulation and improved semantic discrimination also resulted in faster foot responses to hand verbs, thus impairing priming on reaction time. One possible explanation is that reduced activation of the hand-related motor cortex liberated the foot motor cortex from lateral inhibition^65,66^. Also, results show a significant inverse relationship between semantic discrimination and response latency: while a causal relationship cannot be directly deduced, increased verb categorization accuracy accelerating responses appears a more plausible interpretation than shorter response latencies improving semantic accuracy. Faster foot responses to hand verbs may thus depend on a combination of beneficial cathodal stimulation effects on hand verb processing and improved semantic discrimination performance.

Effects of transcranial direct current stimulation are highly dependent on the state of the subject during stimulation^67^. It has been proposed that tDCS interacts with the level of excitation of the system, driven by the task to shape the final result^37^. The present paradigm required discriminating between concrete and abstract verbs, which likely implicates internal simulation of motor behaviour, as confirmed by the presence of priming. Enhanced cortical motor recruitment for concrete compared to abstract verbs^9,22,24,68^ hints at stronger simulation processes accompanying the former. Improved semantic discrimination between concrete and abstract verbs may thus reflect inherent enhanced simulation. Although it may be argued that other parameters can influence discrimination accuracy (e.g., attention), it cannot be excluded that they do so by modulating simulation processes. Our finding of a dependence of cathodal effects on semantic discrimination suggests that simulation needs to be strong enough to allow down-modulation. Interestingly, cathodal tDCS has been shown to decrease MEP amplitude of about 30% at rest and of 50% during motor imagery^69^. Also, cathodal stimulation of the left motor cortex improved coherent motor perception in a complex but not in a simple movement perception condition^36^. These task conditions likely differ in amount of cortical motor recruitment. Altogether, enhanced cortical activation induced by stronger simulation processes (i.e., better versus worse semantic discrimination) may thus boost the effect of cathodal stimulation.

Cathodal stimulation eliminated priming in accuracy measures and, concomitantly to improved semantic discrimination, impaired it on reaction times (compare p = 0.023 in the high d’ subset model versus p < 0.001 in the main model). Dissociation between the impact of stimulation on response accuracy and latency emerged also in other studies focussing on action-related words. Cortical motor cathodal stimulation reduced accuracy of associations between novel words and the appropriate action-related information, whereas it had no impact on reaction time^33^. Cortical premotor repetitive TMS eliminated semantic priming on accuracy for hand verbs while it only reduced priming on reaction time^70^. While these results suggest stronger susceptibility of accuracy measures of priming to neuromodulation, the reasons for that remain to be determined.

No effect of anodal compared to sham stimulation was observed. The polarity effect of tDCS on direct cortical excitability measures like MEP amplitudes appears to fade on behavioural measures^71^. The presence of cathodal but not of opposite anodal effects was previously reported in action word learning^33^ and motor imagery^69^ using the same electrode montage as in the present study. One reason for the lack of anodal influence in the current study may be an induced ceiling effect in cortical excitability preventing further modulation by cognitive processes. The interplay between tDCS and a linguistic task may affect performance measures in a different and more complex way than cortical stimulation alone, thus disrupting tDCS polarity effects.

One limitation of the present study is the lack of objective measures such as MEPs, which renders the interpretations concerning the state of cortical excitability speculative; MEP recording, however, would have not been possible without interfering with the tDCS protocol. Also, tDCS over the foot motor cortex might have well complemented the present double-dissociation paradigm, although at the cost of a resulting 5-way interactions with difficult interpretability. While a more homogenous right-foot lateralization across subjects would have been desirable, the inclusion of response effector as random slope per subject in the mixed linear model (see Methods section) in part mitigates this as it allows the effect of response effector to vary across subjects. Finally, while the categories of hand and foot verbs were matched according to length, frequency, imageability and familiarity, the abstract verb category could not be completely matched except for familiarity. Although this might imply that the effects found are not unequivocally dependent on performance in semantic discrimination, Supplementary Fig. S1 suggests that neither word frequency nor word length were reliable criteria for the distinction between abstract and concrete verbs, which argues for the adequacy of the semantic discrimination task design.

To conclude, the present study shows that cathodal stimulation of the hand-related motor cortex can specifically modulate hand-verb processing, whereby the strength of internal simulation processes appears to further affect this modulation. Verbal-motor interaction has typically been investigated by means of tasks focussing on the lexical/literal/syllabic versus semantic/syntactic dimension. However, our results hint at action comprehension being not an all-or-none phenomenon^72^ and point to the determinant role of an additional dimension such as semantic processing depth. Assessing individual effective motor simulation may result in finer tests of linguistic grounding theories and may provide information on the impact, rather than complementarity, of motor resonance on linguistic understanding. Facilitatory effects of tDCS, which is not aimed at virtually lesioning the cortex, are indicative here and possibly applicable to aphasia^73^ in a supportive and preventive therapeutic approach.

## Electronic supplementary material

Supplementary Information
