# Supplementary material for: Semantic discrimination impacts tDCS modulation of verb processing
Source: Sci Rep. 2017 Dec 7;7:17162. doi: 10.1038/s41598-017-17326-w (PMC5719444; doi:10.1038/s41598-017-17326-w)
Supplement: Supplementary file 1 — Supplementary Information [file 41598_2017_17326_MOESM1_ESM.pdf]

Semantic discrimination impacts tDCS modulation of verb processing

Valentina Niccolai, Anne Klepp, Peter Indefrey, Alfons Schnitzler & Katja Biermann-Ruben

Supplementary Figure

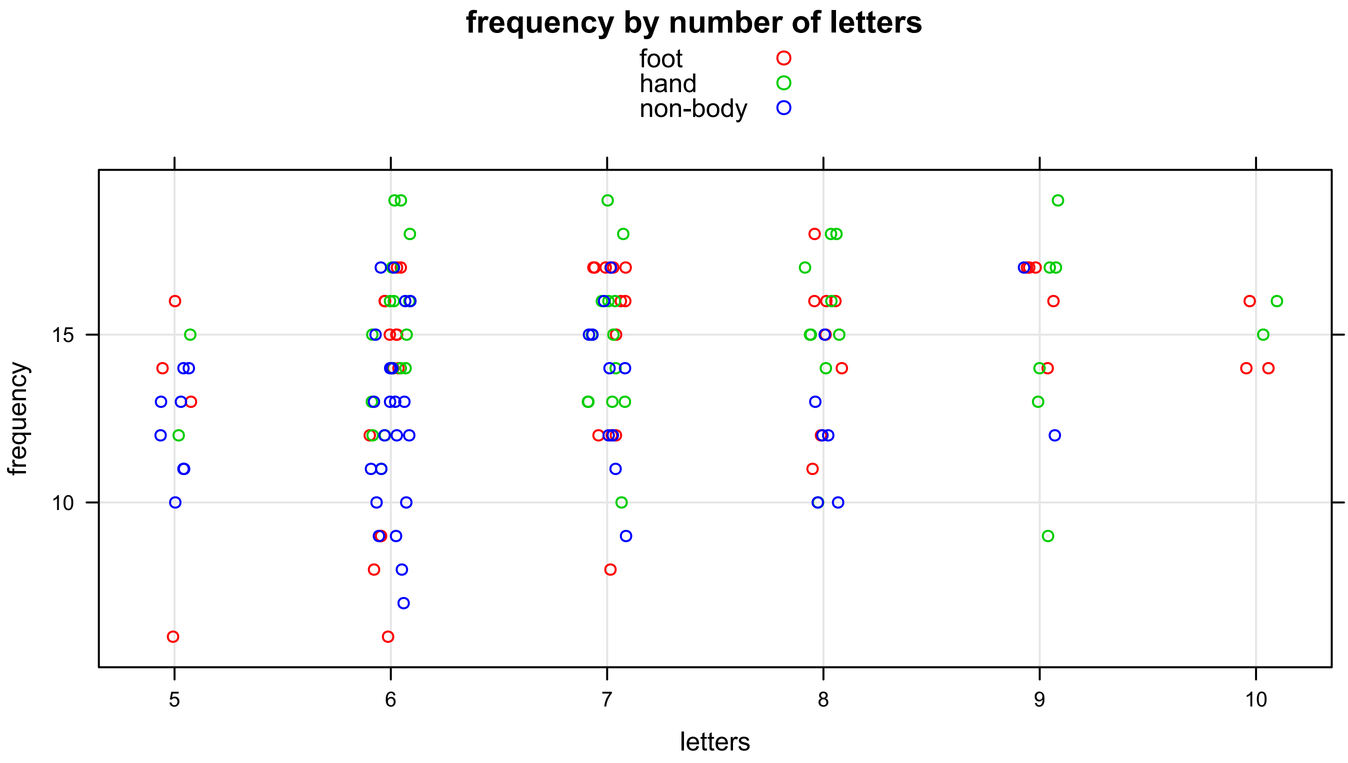

S1. Scatterplot depicting the stimuli from the three verb conditions (foot, hand, and non-body verbs) across the levels of word frequency and length. For visual clarity, data are plotted with a jitter on the x-axis to avoid point overlay.
